# Supplementary material for: Body dissatisfaction widens the racial disparities of Benzophenone-3, a chemical biomarker of personal care and consumer product usage
Source: medRxiv. 2024 Aug 26:2024.08.26.24312258. Preprint. [Version 1] doi: 10.1101/2024.08.26.24312258 (PMC11383470; doi:10.1101/2024.08.26.24312258)
Supplement: Supplement 2 [file media-2.docx]

Body dissatisfaction widens the racial disparities of Benzophenone-3, a chemical biomarker of personal care and consumer product usage

Vy Kim Nguyen^1^, Samuel Zimmerman^2^, Justin Colacino^3^, Olivier Jolliet^4^, Chirag Patel^1^

Affiliations:

^1^Harvard Medical School, Harvard University, Boston, Massachusetts, USA

^2^Broad Institute of Massachusetts Institute of Technology and Harvard, Cambridge, Massachusetts, USA

^3^School of Public Health, University of Michigan, Ann Arbor, Michigan, USA

^4^Technical University of Denmark, Lyngby, Denmark

**Figure S1**. Schematic description of workflow on inclusion criteria of participants and of the statistical methods used to characterize the impact of body dissatisfaction on the racial disparities in biomarker levels of BP3. Models (1)-(2) and (4)-(5) are conducted on the NHANES women population. Models (3) and (6) are conducted specifically in non-Hispanic Black women, while Models (7) and (8) are conducted for each race.


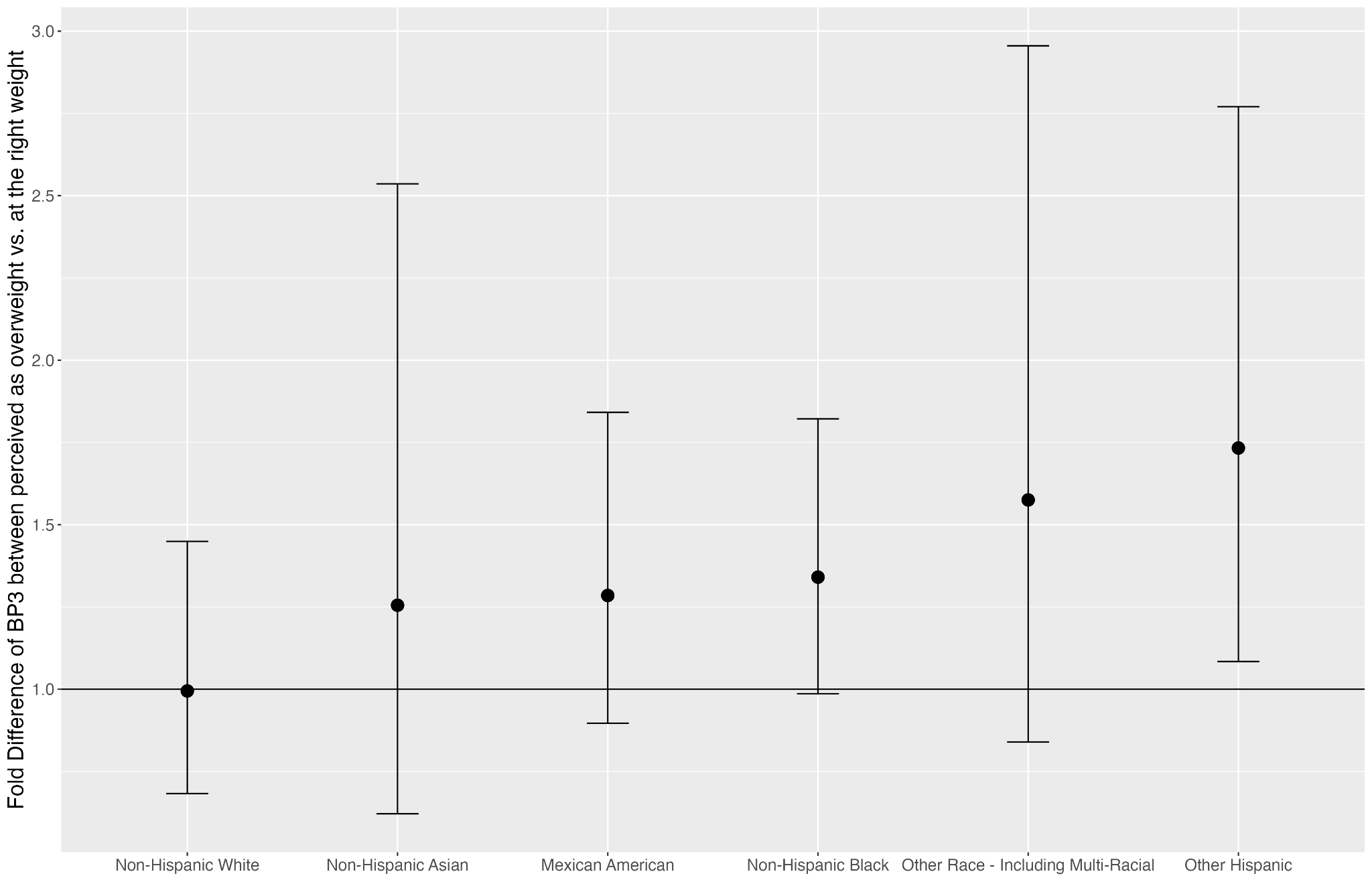


**Figure S2**. Forest plot of fold difference of BP3 biomarker levels between women who perceived themselves as overweight vs. those who perceived themselves at the right weight, **additionally adjusted for sunscreen usage**. The results are from the stratified analyses by race/ethnicity. The reference group is women who perceived themselves at the right weight in a given race/ethnicity. The results are adjusted for age, NHANES cycle, body mass index (BMI), poverty income ratio (PIR), and sunscreen usage.
